# The impact of delayed retirement on the financial sustainability of China’s urban employee pension insurance fund

**DOI:** 10.3389/fpubh.2026.1895268

**Published:** 2026-08-03

**Authors:** Fanqi Wei, Mengru Ji

**Affiliations:** School of Philosophy and Social Sciences, Jilin University, Changchun, China

**Keywords:** actuarial simulation, delayed retirement, financial sustainability, national pooling, urban employee pension insurance fund

## Abstract

Against the backdrop of accelerating population aging and mounting concerns over the financial viability of public pension systems, this paper develops an actuarial simulation framework that integrates demographic dynamics, fund revenue and expenditure rules, and the central adjustment mechanism. We evaluate four scenarios: baseline, delayed retirement only, national pooling only, and the combined reform. Our findings indicate that in the absence of policy intervention, regional disparities in fund balances intensify markedly: the western region enters cumulative deficit first, followed by the central region, while the eastern region retains a longer but still finite reserve buffer. The delayed-retirement-only path does not by itself improve fiscal performance in the present calibration, whereas national pooling narrows current deficits and postpones reserve depletion in deficit regions. When the two reforms are implemented jointly, the combined scenario delivers the strongest overall performance and produces a positive interaction effect on both current and cumulative balances across all three regions. Sensitivity tests further show that pessimistic macro conditions, weaker collection, and lower fund return materially erode the gains, while province-level results reveal substantial heterogeneity within each region. To strengthen the long-run sustainability and institutional resilience of the urban employee pension insurance system, we recommend refining the design of the national pooling mechanism, advancing delayed retirement together with complementary labor-market measures, and adopting regionally differentiated implementation and dynamic adjustment.

## 1 Introduction

Population aging represents one of the most formidable challenges confronting social security systems worldwide, and its implications for the financial sustainability of public pension programs have become increasingly urgent. China stands at the epicenter of this demographic transformation. The country transitioned into an aging society in 1999, entered a deeply aging society by 2021, and is projected to reach super aging status by 2032 (1). The old age dependency ratio of the urban employee basic pension insurance system has deteriorated sharply, declining from 3.2 employed contributors per retiree in 2010 to merely 2.6 in 2024. Simultaneously, the 2019 Comprehensive Plan for Reducing Social Insurance Premium Rates reduced the employer contribution rate from 20 to 16%, further constraining the revenue base of pension funds. Absent decisive policy intervention, actuarial projections indicate that the urban employee pension fund will begin to incur cumulative deficits as early as 2025, with the deficit potentially reaching catastrophic proportions by midcentury (2). The confluence of rapidly aging demographics and declining contribution revenues has thus elevated pension sustainability from a long-term fiscal concern to an immediate policy imperative.

In response to these mounting pressures, the Chinese government has initiated two landmark reforms. First, a national pooling (central adjustment) mechanism for basic pension insurance funds was implemented in 2022, designed to reallocate resources from surplus provinces to deficit provinces and thereby mitigate the severe regional imbalances that characterize China’s fragmented pension system. Second, effective January 1, 2025, China commenced a gradual postponement of the statutory retirement age. Under this reform, the retirement age for male workers will rise from 60 to 63, for female cadres from 55 to 58, and for female workers from 50 to 55, phased in over a 15-year period. Additionally, starting from January 1, 2030, the minimum contribution period for pension eligibility will increase incrementally from 15 to 20 years. These two policies—national risk pooling and retirement age extension—operate through distinct mechanisms: the former addresses spatial imbalances across regions, while the latter targets intertemporal imbalances between contributions and benefits (3). Understanding how these policy instruments jointly affect the financial trajectory of China’s pension system constitutes a question of first order importance for both academic research and policy design.

A substantial body of research has examined the financial sustainability of pension systems in aging societies. One strand of the literature focuses on parametric reforms, including adjustments to contribution rates, benefit formulas, and eligibility ages. Costrell and McGee provide a theoretical framework formalizing pension sustainability through the lens of steady state contribution rates (4), while Devriendt et al. demonstrate, using an overlapping generations model with heterogeneous agents, that raising the normal retirement age can restore financial equilibrium but may exacerbate intergenerational and intragenerational welfare inequality (5). Sánchez Romero et al. further show that retirement age increases, although favorable for aggregate macroeconomic outcomes, tend to increase inequality across socioeconomic groups when life expectancy and labor income are heterogeneous (6). On the empirical side, Dolls and Krolage exploit a German pension reform to demonstrate that changes in the full retirement age exert substantial effects on claiming behavior (7), while Gruber et al. document that even the relabeling of retirement ages without changes in financial incentives can shift retirement decisions through reference point effects (8). A broader perspective on the trajectory of pension reform is provided by Hinrichs, who argues the cumulative effect of reduced accrual rates, the widespread shift from wage to price indexation, and the tightening of early retirement pathways has systematically eroded the poverty prevention function of pension systems (9). Ebbinghaus demonstrates that while pension systems compress income inequality relative to working age populations, their earnings-related design simultaneously reproduces labor market ‘double-edged income effect’ that is especially pronounced in countries that have shifted toward multipillar architectures with substantial private, funded components (10). Using a general equilibrium overlapping generations model calibrated to Taiwan, Lin illuminates a fundamental tension between fiscal efficiency and macroeconomic performance in structural pension reform (11). Zhang conducted a study on the optimal retirement age in China and found that a gradual delay scheme can effectively alleviate the fund payment pressure and reduce long-term actuarial deficits (12).

A second strand of research specifically addresses pension reform in the Chinese context. Li and Cang find that better health delays retirement in studying China’s pension system, while higher pension replacement ratios encourage earlier exit, especially for low-income workers, with health effects operating through income and risk channels that asymmetrically impact unhealthy versus healthy households (13). Wang contends that integrating the delayed retirement policy with policy adjustments, such as changes in contribution rates, can achieve a more favorable trade-off between pension adequacy and fiscal sustainability, while also underscoring the importance of adopting an elastic retirement mechanism (14). Wu et al. use an overlapping generations framework to compare voluntary versus mandatory delayed retirement in China, concluding that voluntary schemes are superior for both fund sustainability and retiree welfare (15). Zhao et al. construct an actuarial income–expenditure model and project that under the existing system, China’s basic pension insurance fund will encounter its first deficit in 2027, while delayed retirement postpones this to 2029 but cannot fully close the gap (16). Chen and Shang focus on the interaction between contribution rate reductions and national pooling, showing that the latter can sustain long-run solvency even at a reduced 16% contribution rate, with insolvency postponed to 2,113 when collection responsibility is transferred to taxation authorities (2). These studies collectively underscore that isolated policy instruments are insufficient to address the systemic challenge posed by population aging.

A third strand of the literature examines how pension systems interact with large-scale economic shocks. Bottan et al. demonstrate that preexisting pension infrastructure can function as an automatic stabilizer during systemic shocks, delivering welfare protections comparable to the combined effect of income targeted cash transfers and unemployment insurance (17). Lorca analyzed Chile’s temporary policy that permitted individuals to withdraw up to 10 % of their private pension savings during the pandemic, and provided it exacerbating retirement income inequality and increasing fiscal pressure on the public safety net. A fourth and emerging strand of scholarship addresses the governance and investment dimensions of pension fund management (18). Dana argues that statutory reform, rather than fluctuating administrative guidance, is necessary to provide stable direction that enables pension fiduciaries to incorporate environmental, social, and governance considerations without breaching their fiduciary duty of loyalty (19).

Despite these important contributions, three specific gaps remain in the existing literature. First, although separate studies have examined either national pooling or delayed retirement, the interactive and potentially synergistic effects of their joint implementation remain substantially underexplored. Prior work tends to evaluate individual instruments in isolation, leaving open the question of whether and through which channels policy combination generates effects that differ from the sum of individual impacts. Second, the pronounced regional heterogeneity embedded in China’s pension system, which driven by divergent provincial demographic profiles, wage levels, and historical fund accumulations, is frequently obscured by national level or single province analyses. Studies that aggregate to the national level cannot capture how the same national policy produces markedly different fiscal outcomes across the eastern, central, and western regions (14, 15). Third, with the notable exception of a few recent contributions, the bulk of the existing simulation literature relies on hypothetical retirement age trajectories or proposed reform blueprints rather than the specific phased in parameters legislated in China’s 2025 reform package. As a result, the quantitative projections available to date may not adequately inform the ongoing calibration of the reform as actually enacted.

This paper addresses these gaps by constructing a province level actuarial simulation framework that jointly integrates population dynamics, fund revenue and expenditure rules, and the central adjustment mechanism. Drawing on provincial level statistical data from 2020 to 2023 as the calibration baseline, we simulate the financial trajectory of China’s urban employee basic pension insurance fund from 2024 to 2050 under four sequentially nested policy scenarios: (a) a no intervention baseline, (b) delayed-retirement-only, (c) the national pooling (central adjustment) mechanism as implemented in 2022, and (d) the synergistic combination of national pooling with the gradual delayed retirement reform that took effect on January 1, 2025. Critically, scenario (d) incorporates the legislated reform parameters—the statutory retirement age rising from 60 to 63 for males, 55 to 58 for female cadres, and 50 to 55 for female workers over a 15-year phase in, together with the increase in the minimum contribution period from 15 to 20 years starting in 2030. The provincial granularity of the model enables us to decompose the differential impacts of these policy instruments on fund revenue, expenditure, current balances, and cumulative reserves across China’s eastern, central, and western regions throughout the projection horizon.

This study makes three principal contributions. First, we quantify the synergy between national pooling and delayed retirement within a unified provincial level framework, decomposing their combined effect into spatial and temporal channels. We show that national pooling predominantly attenuates cross provincial fiscal disparities through resource redistribution, while delayed retirement improves the intertemporal balance by simultaneously broadening the contribution base and deferring benefit claims. Their joint effect on cumulative fund balances exceeds the additive sum of individual policy effects, a finding with direct implications for the sequencing and coordination of pension reform. Second, by conducting the entire analysis at the provincial level and explicitly modeling the central adjustment formula, we provide a regionally disaggregated picture of how identical national policies generate heterogeneous fiscal outcomes depending on each province’s demographic structure, wage level, and initial fund position. This granularity is essential for designing equitable transition arrangements and targeted supplementary measures. Third, we anchor the long-run fund simulations in the actual legislated parameters of China’s 2025 gradual delayed retirement reform—including the occupation specific retirement age schedules, the 15-year phase in period, and the increase in the minimum contribution requirement, rather than in hypothetical policy designs. This ensures that our projections speak directly to the reform trajectory China has already committed to and provides a quantitative benchmark against which future policy adjustments can be evaluated.

The remainder of the paper is structured as follows. Section 2 presents the actuarial model and parameter specifications. Section 3 describes the data sources and presents the numerical simulation results under the four policy scenarios. Section 4 provides an in-depth analysis and discussion of the mechanisms through which national pooling and delayed retirement affect fund sustainability, including their synergistic effects and practical limitations. Section 5 concludes with policy recommendations and directions for future research.

## 2 Model specification and parameter calibration

This paper takes the provincial level as the unit of analysis, integrating multiple variables including population structure, wage growth trajectories, and institutional parameters to construct province specific pension fund revenue and expenditure models alongside the central adjustment mechanism framework. The basis for setting key parameters is explained in detail below.

The model is constructed around four guiding principles: (1) Institutional Consistency. All parameters and formulas are specified in strict accordance with national policy documents to ensure the model’s operational validity and comparability with real world institutional arrangements. (2) Population Dynamics. Changes in fund revenue and expenditure are fundamentally driven by demographic structure. Accordingly, the cohort component method is employed to compute the number of employed and retired individuals in each age group, thereby capturing the dynamic transmission of population changes to fund flows. (3) Behavioral Responsiveness. The delayed retirement policy alters individuals’ retirement behavior, which in turn affects the contribution period, the accumulation in personal accounts, and current expenditures. These channels are explicitly incorporated into the model. (4) Regional Differentiation. The national pooling and central adjustment system is designed to mitigate regional disparities. The model therefore simulates revenue–expenditure gaps and adjustment flows at the provincial level to capture cross regional heterogeneity.

### 2.1 Province level revenue and expenditure models

In accordance with the relevant provisions of Chinese social insurance law and building on mainstream pension actuarial approaches (3, 16), we construct an actuarial projection model that captures the impact of retirement age adjustments on fund revenue and expenditure.

#### 2.1.1 Pension fund revenue model

Article 12 of the Social Insurance Law of the People’s Republic of China stipulates that employees and employers shall jointly contribute to the basic pension insurance fund based on the statutory contribution base and contribution rate. The actual amount deposited must account for incomplete collection, and is thus multiplied by the collection rate. Accordingly, the pension insurance fund revenue of each province is expressed as: the number of insured employed workers × the contribution base × the contribution rate × the collection rate, as specified in Equation 1.


$${\left(AC\right)}_{t}^{i}=\overset{3}{\underset{j=1}{\sum }}\overset{b_{j}-1}{\underset{x=a_{j}}{\sum }}N_{t,x}^{i,j}\cdot \omega_{t}^{i}\cdot \left(R_{1}+R_{2}\right)\cdot \alpha$$ (1)

$$\begin{array}{c} \omega_{t}^{i}=\varphi_{t_{0}}^{i}\cdot \prod_{s=t_{0}+1}^{t}\left(1+k_{s}^{i}\right) \end{array}$$

where ${\left(AC\right)}_{t}^{i}$ denotes the total pension insurance fund revenue of province i in year t ; j=1,2,3 represent male officials, female officials, and female workers respectively; $a_{j}$ is the initial insurance enrollment age (set to 20 following standard practice); $b_{j}$ is the statutory retirement age for worker category j ; $N_{t,x}^{i,j}$ is the number of insured employed workers of type j at age x in province i in year t ; $\omega_{t}^{i}$ is the contribution base of province i in year t ; $R_{1}=16\%$ and $R_{2}=8\%$ are the employer and individual contribution rates, respectively; α is the collection rate (national average approximately 90–95%, and it is set at 93%); $t_{0}$ denotes the starting year of the actuarial projection; $\varphi_{t_{0}}^{i}$ is the average wage of all employed persons in urban units of province i in the base year; and $k_{s}^{i}$ is the average wage growth rate of province i in year s .

#### 2.1.2 Pension fund expenditure model

The basic pension benefit is calculated as: (provincial average social wage + personal indexed average contribution wage) ÷ 2 × contribution years × 1%. The personal indexed average contribution wage equals the contribution base multiplied by the personal average contribution wage index, where the average contribution index is the ratio of the individual contribution wage to the average social wage. Because it is empirically infeasible to reconstruct each individual’s contribution history, we follow the established actuarial convention of approximating the contribution base by the provincial macro-average social wage (3, 16), which implies an average contribution index of approximately unity. Under this approximation, the indexed average contribution wage converges to the average social wage in the long run, and both the contribution base and the average social wage can be treated as roughly equivalent. Consequently, basic pension expenditure simplifies to Equation 2.


$$\begin{array}{c} Q_{t}^{i}=\overset{3}{\underset{j=1}{\sum }}\overset{c}{\underset{x=b_{j}}{\sum }}N_{t,x}^{i,j}\cdot \mu_{t}^{i}\cdot \left(b_{j}-a_{j}\right)\cdot \beta \cdot \prod_{s=t-\left(x-b_{j}\right)+1}^{t}\left(1+g_{s}^{i}\right) \end{array}$$ (2)

$$\begin{array}{c} \mu_{t}^{i}=\mu_{t_{0}}^{i}\cdot \prod_{s=t_{0}+1}^{t-\left(x-b_{j}\right)-1}\left(1+h_{s}^{i}\right) \end{array}$$

where $Q_{t}^{i}$ denotes total basic pension expenditure of province i in year t ; c is the maximum attainable age (set to 100); $\mu_{t}^{i}$ is the benefit calculation base for province i in year t , approximated by the provincial average social wage; β is the basic pension accrual rate (1% per year of contribution as stipulated by the Social Insurance Law); $t-\left(x-b_{j}\right)$ is the statutory retirement age for worker category j ; $g_{s}^{i}$ is the annual pension benefit growth rate (calibrated to approximately 5% based on average national adjustment rates over 2020–2023); and $h_{s}^{i}$ is the growth rate of the benefit calculation base, which tracks the nominal wage growth rate.

The transitional pension compensates for the benefit gap created by the 1997 pension system reform. It covers employees who were insured prior to the reform, had not yet retired at the time of the reform, and retired thereafter. In line with official regulations, the transitional pension is computed as Equation 3.


$$\begin{array}{c} \begin{array}{l} W_{t}^{i}=\overset{3}{\underset{j=1}{\sum }}\overset{\min\left\{t-z+b_{j},c\right\}}{\underset{x=\max\left\{b_{j},t-z+a_{j}\right\}}{\sum }}N_{t,x}^{i,j}\cdot \mu_{t}^{i}\\ \cdot \left[z-\left(t-x+a_{j}\right)\right]\cdot \varepsilon \cdot \prod_{s=t-\left(x-b_{j}\right)+1}^{t}\left(1+g_{s}^{i}\right) \end{array} \end{array}$$ (3)

where $W_{t}^{i}$ denotes transitional pension expenditure of province i in year t ; z is the year of the pension system reform that introduced the unified basic pension framework; $t-z+a_{j}t$ is the age of insured employees at the time of reform who are still alive at the end of year t ; $t-z+b_{j}$ is the age of those already retired at the reform year who survive to year t ; $z-\left(t-x+a_{j}\right)$ measures pre-reform contribution years; and ε is the transitional coefficient set in accordance with provincial implementation guidelines.

The Social Insurance Law further stipulates that the basic pension consists of the basic pension component and the individual account pension. The monthly individual account pension equals the accumulated individual account balance divided by the number of prescribed distribution months. To simulate the annual accumulation of individual account savings, we derive the following expression presented in Equation 4.


$$\begin{array}{c} \begin{array}{l} E_{t}^{i}=\overset{3}{\underset{j=1}{\sum }}\overset{c}{\underset{x=b_{j}}{\sum }}\left[N_{t,x}^{i,j}\cdot \frac{\begin{array}{l} \sum_{s=\max\left\{t-\left(x-a_{j}\right)-1,z\right\}}^{t-\left(x-b_{j}\right)-1}\\ \omega_{s}\cdot R_{2}\cdot {\left(1+r\right)}^{t-\left(x-b_{j}\right)-s} \end{array}}{m_{j}}\cdot 12\right]\\ \cdot \prod_{s=t-\left(x-b_{j}\right)+1}^{t}\left(1+g_{s}^{i}\right) \end{array} \end{array}$$ (4)

where $E_{t}^{i}$ denotes personal account pension expenditure of province i in year t ; $t-\left(x-a_{j}\right)$ is the year of initial insurance enrollment; r is the personal account notional interest rate (set to the 1 year bank deposit rate plus a spread, averaging approximately 2.5–3.0% over the projection period); $\sum_{s=\max\left\{t-\left(x-a_{j}\right)-1,z\right\}}^{t-\left(x-b_{j}\right)-1}\omega_{s}\cdot R_{2}\cdot {\left(1+r\right)}^{t-\left(x-b_{j}\right)-s}$ is the cumulative individual account balance of insured workers of type j at age x in province i at the beginning of year t ; and $m_{j}$ is the prescribed number of distribution months for category j (139 for males retiring at 60, 170 for females retiring at 55, and 195 for females retiring at 50, with proportional adjustments under the delayed retirement schedule).

Aggregating the three components above, the total expenditure of the pension insurance fund is given by Equation 5.


$$\begin{array}{c} {\left(AE\right)}_{t}^{i}=Q_{t}^{i}+W_{t}^{i}+E_{t}^{i} \end{array}$$ (5)

#### 2.1.3 Accumulated reserve model

The cumulative balance of the pension insurance fund in a given year comprises the cumulative balance carried forward from the previous year plus the current year balance, adjusted for investment income. Since provincial pension balances earn interest, this income must be incorporated. The current year balance is the net amount obtained by subtracting total expenditure from total revenue. The detailed computation is given in Equation 6.


$$\begin{array}{c} F_{t}^{i}=\left(F_{t-1}^{i}+S_{t}^{i}\right)\cdot \left(1+i_{t}\right)=\left\{F_{t-1}^{i}+\left[{\left(AC\right)}_{t}^{i}-{\left(AE\right)}_{t}^{i}\right]\right\}\cdot \left(1+i_{t}\right) \end{array}$$ (6)

where $F_{t}^{i}$ denotes the accumulated balance of the pension insurance fund of province i at the end of year t ; $S_{t}^{i}$ is the current year surplus (revenue minus total expenditure) before central adjustment; and $i_{t}$ is the fund preservation and appreciation rate, calibrated to the average yield on provincial pension fund deposits and government bond portfolios, approximately 3.0–4.5% per annum depending on the province.

### 2.2 Central adjustment mechanism for pension insurance funds

The central adjustment mechanism is an institutional arrangement for balancing the fiscal burden of pension insurance funds across provinces and safeguarding the stable operation of the national pension system. Its core logic is a central level fiscal transfer: funds are collected from provinces with relatively ample surpluses and redistributed to provinces facing significant deficits, thereby operationalizing cross regional risk sharing. We model this mechanism based on the official policy framework.

#### 2.2.1 Revenue collection

The central adjustment fund is financed by transfers from each province’s pension insurance fund. The transfer amount for a given province is calculated as: (provincial average employee wage × 90%) × the number of employed individuals required to be insured in that province × the transfer ratio. The transfer ratio begins at 3% and is scheduled to increase progressively. The formula is presented in Equation 7.


$$\begin{array}{c} U_{t}^{i}=N_{t,x}^{i,j}\cdot \varphi_{t}^{i}\cdot 90\%\cdot \theta_{t} \end{array}$$ (7)

The aggregate revenue of the national central adjustment fund is expressed by Equation 8.


$$\begin{array}{c} U_{t}^{N}=\overset{N}{\underset{i=1}{\sum }}U_{t}^{i} \end{array}$$ (8)

where $U_{t}^{i}$ denotes the transfer amount from province  i to the central adjustment fund in year t , computed as 90% of the provincial average wage multiplied by the number of employed insured persons and the nationally uniform allocation ratio $\theta_{t}$ . The ratio $\theta_{t}$ starts at 3.0% in 2022 and is scheduled to increase progressively; for the simulations we set $\theta_{t}$ = 3.5% from 2025 onward, consistent with policy guidance.

#### 2.2.2 Fund allocation

The central adjustment fund operates on the principle that revenue determines expenditure: all funds raised in a given year are fully allocated to the provinces within the same year. Disbursement follows a fixed per capita quota. The allocation to each province is determined by the number of verified retirees in that province, as jointly certified by the Ministry of Human Resources and Social Security and the Ministry of Finance, multiplied by the national average per capita allocation amount. The formula is given in Equation 9.


$$\begin{array}{c} D_{t}^{i}=L_{t}^{i}\cdot v_{t} \end{array}$$ (9)

The national average allocation amount is defined as the total central adjustment fund raised divided by the nationally verified total number of retirees. The specific formula appears in Equation 10.


$$\begin{array}{c} v_{t}=\frac{U_{t}^{N}}{L_{t}} \end{array}$$ (10)

where $D_{t}^{i}$ is the allocation amount received by province i in year  t ; $L_{t}^{i}$ is the number of verified retirees in province i in year  t , as certified jointly by the Ministry of Human Resources and Social Security and the Ministry of Finance; $v_{t}$ is the national per capita allocation amount; and $L_{t}$ is the total number of nationally verified retirees.

It follows directly that:


$$\begin{array}{c} \overset{N}{\underset{i=1}{\sum }}D_{t}^{i}=L_{t}\cdot v_{t}=L_{t}\cdot \frac{U_{t}^{N}}{L_{t}}=U_{t}^{N} \end{array}$$ (11)

Equation 11 embodies the defining feature of the central adjustment fund: all resources raised in the current year are fully disbursed within the same year, ensuring an exact annual balance between revenue and expenditure at the aggregate level.

#### 2.2.3 Post allocation provincial balances

The central adjustment system mandates that all funds transferred from provinces to the central government be reallocated to deficit provinces within the same fiscal year. Accordingly, for each province, the current year balance is adjusted by subtracting the transferred amount which treated as an expenditure and adding the allocation received which treated as revenue. The post adjustment current balance is given by Equation 12.


$$\begin{array}{c} S_{t}^{i^{\prime }}=S_{t}^{i}-U_{t}^{i}+D_{t}^{i} \end{array}$$ (12)

The parameter specifications follow the principle that institutional parameters are aligned with policy documents, economic parameters are calibrated to statistical data, and behavioral parameters are set to literature averages, thereby ensuring that the model maintains both policy consistency and real world comparability.

The cumulative balance captures the long-run financial sustainability of the fund. In accordance with the fund management regulations, each province’s cumulative balance equals its prior year cumulative balance plus the post adjustment current balance plus investment returns from fund preservation and appreciation, as shown in Equation 13.


$$\begin{array}{c} F_{t}^{i^{\prime }}=\left(F_{t-1}^{i}+S_{t}^{i^{\prime }}\right)\cdot \left(1+i_{t}\right) \end{array}$$ (13)

In the formula, $S_{t}^{i^{\prime }}$ represents the current balance after adjustment in province i for the t year period, $F_{t}^{i^{\prime }}$ represents the cumulative balance after adjustment in province i over t years.

Equation 13 illustrates the transmission channel through which the central adjustment system shapes long-run fund dynamics: the adjustment alters the current surplus, and the revised surplus continues to compound through interest accruals, thereby exerting a persistent influence on the intertemporal trajectory of fund balances.

### 2.3 Parameter specification

The parameter specifications adhere to the following principle: institutional parameters are drawn from official policy documents, economic parameters are calibrated to statistical data, and behavioral parameters are set to the central tendencies reported in the literature. This approach ensures that the model maintains both policy fidelity and empirical comparability. The specific parameter configurations are presented in Table 1.

**Table 1:** Parameter settings.

Parameters	Meaning	Value
$R_{1}$	Unit contribution rate	16%
$R_{2}$	Individual contribution rate	8%
$\omega_{t}^{i}$	The contribution base for year t in Province i	Equal to the average salary of all employees in urban units in the previous year (including all forms of compensation)
α	Collection rate	The national average is approximately 90–95%, and it is set at 93%.
$k_{s}^{i}$	The average growth rate of total wages in Province i for the year s	Calculate the average based on the growth rates over the years.
$g_{s}^{i}$	The growth rate of pensions in Province i for the year s	Calculate the average growth rate based on the annual growth rates of pensions over the years.
$i_{t}$	The rate of preservation and appreciation of the pension fund for the year t	The actual return rate of local pension investments has been in the range of 4–6% in recent years. It is set at 5%.
c	Maximum lifespan	The average life expectancy is set at 79 years; the maximum survival age in the model is set at 100 years.
$b_{j}$	The legal retirement age	The age range for men gradually increased from 60 to 63; for female cadres and female workers, it gradually changed from 55/50 to 58/55.
$N_{t,x}^{i,j}$	The number of insured employed workers of type j at age x in province i in year t	Based on the data from the seventh national census in 2020, predictions were made using the cohort approach.
$t_{0}$	The starting year for the actuarial analysis	2024
$a_{j}$	Initial enrollment age	22 years old
$\varphi_{t_{0}}^{i}$	The average wage of all employed persons in urban units of province i in the base year	Obtained from the statistical yearbooks of each province
$\mu_{t}^{i}$	The base salary amount for province i	Equal to the average salary of all employees in urban units in the previous year (including all forms of compensation)
β	Pension payment ratio	1%
z	Year of the reform of the old-age insurance system	1997

The principal policy documents consulted include: the Central Adjustment System for Enterprise Employees’ Basic Pension Insurance Fund; the Comprehensive Plan for Reducing Social Insurance Premium Rates; the Gradual Postponement of the Statutory Retirement Age; and the Measures for the Gradual Increase of the Minimum Contribution Period for Basic Pension Insurance. Mortality parameters are drawn from the China Resident Life Table. Key literature references for parameter calibration include the steady state contribution rate framework (4), collection rate assumptions (2), demographic response parameters (20), and behavioral elasticity estimates (15).

## 3 Data and numerical simulation

### 3.1 Data sources

The data employed in this study are drawn from national statistical agencies, official provincial government releases, and annual statistical reports published by the Ministry of Human Resources and Social Security. The dataset encompasses population structure, insurance participation status, fund revenue and expenditure, wage levels, and related macroeconomic indicators, providing a robust empirical foundation for model construction and calibration.

For population data, we use the age specific distribution from the Seventh National Population Census in 2020 as the baseline for demographic projections. The census offers comprehensive age detail, broader coverage, and higher statistical precision than annual sample surveys, making it the most reliable benchmark for long-run population simulations. Because pension fund flows are highly sensitive to the age structure, we refine the census’s 5 year age group data into single year age series using the standard proportional decomposition method, thereby constructing the full age distribution required for dynamic demographic forecasting. In addition, we incorporate the China Resident Life Table published by the National Health Commission and apply the cohort component method to project the natural attrition of each age cohort over the simulation horizon, ensuring that the population structure remains dynamically consistent with observed mortality patterns.

Data on insured populations and fund operations are sourced primarily from the annual Statistical Bulletin published by the Ministry of Human Resources and Social Security and the corresponding annual operational reports released by provincial human resources and social security departments. These sources provide key indicators which include the number of insured employed workers, the number of retirees, fund revenues and expenditures, current surpluses, and cumulative balances, for the urban employee basic pension insurance system. We use the provincial fund income, expenditure, and insured population structure for 2020–2023 as reference points, and calibrate the macro average benefit level by back solving from these official statistics, thereby aligning the model’s base period output with the observed fiscal position and anchoring the long-run projections. The participation assumption in the baseline simulation is that insured employment evolves according to province-specific historical growth trends unless altered by the delayed-retirement mechanism.

Wage levels and economic growth parameters are drawn from provincial statistical yearbooks and the national economic operation data released by the National Bureau of Statistics. The average wage of employed persons in urban units serves as the basis for computing both the contribution base and the benefit calculation base, with its growth rate reflecting long-run regional economic trends. For pension indexation, we extract the average adjustment rate from recent national pension adjustment plans and use this as the benchmark input. A sensitivity analysis will be conducted in the subsequent sections.

### 3.2 Historical performance of the pension insurance fund

**Figure 1:**
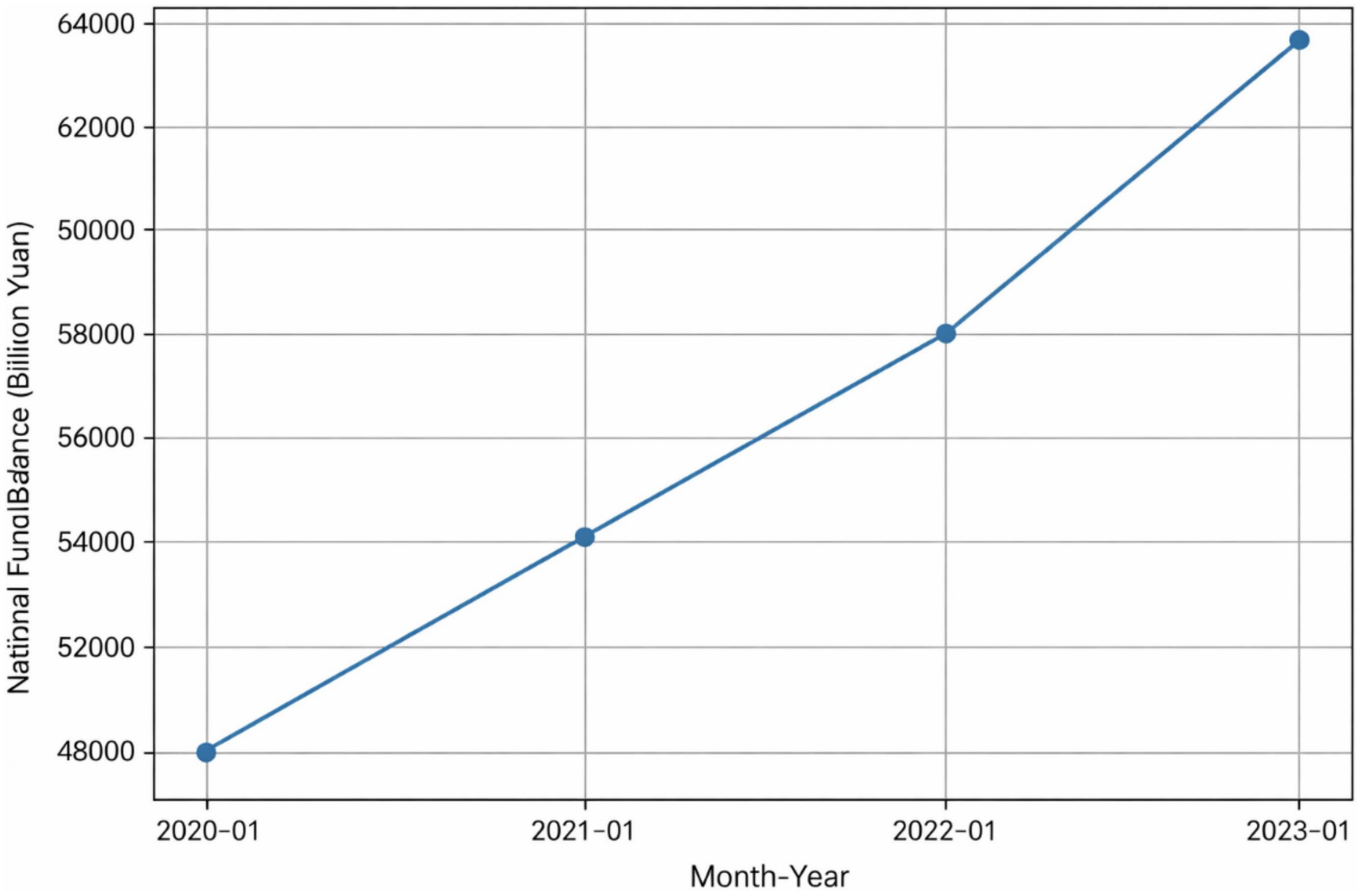
Historical trend of national pension fund surplus changes.

Figure 1 displays the trajectory of the national pension insurance fund’s cumulative balance from 2020 to 2023. Over this period, the cumulative balance exhibited a sustained upward trend, rising from approximately 4.83 trillion yuan in 2020 to roughly 6.37 trillion yuan in 2023, with a cumulative increase of nearly 1.5 trillion yuan. This expansion reflects the combined effects of post pandemic macroeconomic recovery, enhanced fiscal support, and improved investment returns, and indicates that the national fund retains a substantial buffer at the current juncture.

**Figure 2:**
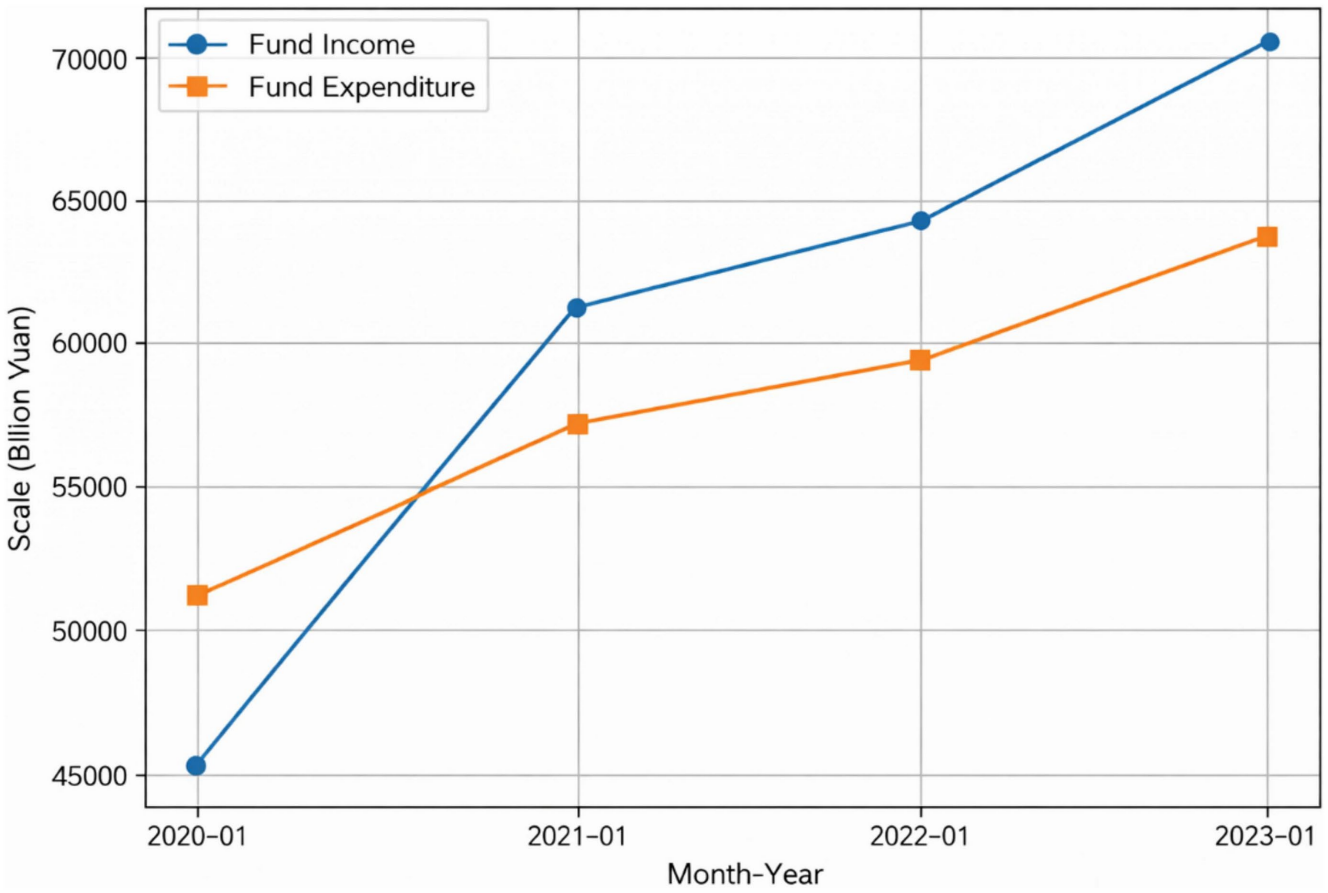
Trend of changes in national pension fund income and expenditure.

Figure 2 plots the synchronous upward trajectories of national pension fund revenue and expenditure over the 2020–2023 period. Fund revenue increased from approximately 4.45 trillion yuan in 2020 to roughly 7.05 trillion yuan in 2023, while expenditure rose from about 5.13 trillion yuan to approximately 6.38 trillion yuan. Notably, the growth rate of revenue outpaced that of expenditure, particularly between 2020 and 2021, which accounts for the sustained expansion of the national cumulative balance. However, the revenue–expenditure gap has not widened continuously, suggesting that the rigidity of pension spending is intensifying as population aging accelerates. This pattern foreshadows growing fiscal pressure on the fund’s future balance.

### 3.3 Numerical simulation

The detailed formulas in Section 2 enter the simulation in a two-step manner. First, the province-level number of contributors and retirees is updated year by year according to demographic aging, retirement-age rules, and insured-employment trends. Second, the basic pension, transitional pension, and individual account pension formulas are aggregated into an annual macro-average benefit level for each province, allowing the model to connect the institutional benefit rules to the observed expenditure totals. Because it is empirically challenging to reconstruct the complete payment history and benefit profiles of each retirement cohort, we adopt the following approximation Equation 14 to implement the expenditure block in the simulation.


$$\begin{array}{c} {\left(AE\right)}_{t}^{i}\approx N_{t}^{i}�P_{t}^{i}\# \end{array}$$ (14)

In this expression, the term denoted by $P_{t}^{i}$ can be interpreted as the macro-average benefit level that is the reduced-form outcome of the benefit calculation base, cumulative contribution years, individual account balances, prescribed distribution months, and annual pension indexation. Delayed retirement affects this term through several channels simultaneously: it reduces the number of new retirees entering the benefit pool, extends contribution years for those who remain employed, enlarges personal account accumulations, and shifts the timing of pension drawdown. We calibrate the macro-average benefit using official base-period statistics so that the model-generated expenditures reproduce the observed fiscal aggregates before the forward projection is carried out.

We construct four policy scenarios. (1) Baseline scenario: all parameters remain at their historical values; the numbers of employed contributors and retirees, as well as wages and pensions, evolve according to their historical average growth rates without additional policy intervention. (2) Delayed-retirement-only scenario: the statutory retirement-age reform is introduced while the pension system remains without national pooling, allowing the stand-alone temporal effect of retirement postponement to be identified. (3) National pooling scenario: each province transfers a prescribed proportion of its contribution revenue to the central adjustment fund, and the pooled resources are distributed uniformly on a per-retiree basis, simulating the redistributive effect of the national pooling system on regional imbalances. (4) Combined scenario: delayed retirement is implemented within the national pooling framework. This four-scenario design permits direct identification of the interaction effect as: combined scenario minus pooling-only scenario minus delayed-retirement-only scenario plus baseline.

The delayed-retirement path is modeled in accordance with the legislated reform timetable. The retirement age for male workers rises from 60 to 63, for female cadres from 55 to 58, and for female workers from 50 to 55, with the adjustment phased in linearly from 2025 through 2039. Starting in 2030, the minimum contribution period for pension eligibility is increased gradually from 15 to 20 years. Workers who remain in the insured labor force because of the higher retirement age continue to contribute, postpone pension claiming, and accumulate larger individual accounts.

#### 3.3.1 Baseline and delayed-retirement-only scenario

Table 2 reports both the benchmark baseline path and the delayed-retirement-only path, allowing the independent contribution of retirement-age postponement to be separated from the redistributive effect of national pooling.

**Table 2:** Baseline and delay retirement scenario (unit: trillion yuan).

Region	Year	Baseline		Delay retirement	
		Current	Cumulative	Current	Cumulative
Eastern region	2025	0.42	4.85	0.41	4.85
	2030	0.18	4.40	−0.01	3.75
	2035	−0.12	3.20	−0.24	1.55
	2040	−0.35	1.35	−0.26	−0.79
	2050	−0.62	−0.80	−1.35	−8.62
Central region	2025	0.05	1.60	0.02	1.56
	2030	−0.18	0.95	−0.32	0.41
	2035	−0.45	−0.20	−0.63	−1.78
	2040	−0.72	−1.80	−0.81	−4.54
	2050	−0.95	−4.10	−1.34	−11.85
Western region	2025	−0.10	0.95	−0.12	0.93
	2030	−0.35	0.20	−0.55	−0.48
	2035	−0.62	−1.50	−0.88	−3.65
	2040	−0.88	−3.80	−1.08	−7.82
	2050	−1.20	−7.50	−1.71	−18.64

Under the baseline path, the eastern region’s current balance falls from 0.42 trillion yuan in 2025 to −0.62 trillion yuan in 2050, the central region declines from 0.05 to −0.95 trillion yuan, and the western region deteriorates from −0.10 to −1.20 trillion yuan. The cumulative balance turns negative first in the western region by 2035, then in the central region by 2035, and finally in the eastern region by 2050, confirming a clear west-center-east gradient in fiscal stress.

The delayed-retirement-only path added in Table 2 is weaker than the baseline in the present calibration. By 2050, current balances reach −1.35, −1.34, and −1.71 trillion yuan in the eastern, central, and western regions, respectively, while cumulative balances decline to −8.62, −11.85, and −18.64 trillion yuan. This result indicates that retirement postponement, when modeled in isolation and without the support of interprovincial redistribution, does not by itself offset the adverse demographic and institutional pressures embedded in the projection.

#### 3.3.2 National pooling scenario

The introduction of the national pooling mechanism markedly improves the provincial distribution of pension fund balances across all regions, as detailed in Table 3. By construction, Equation 11 implies that pooling reallocates resources across provinces but does not create new aggregate national resources.

**Table 3:** National unified planning scenario (unit: trillion yuan).

Region	Indicators	2025	2030	2035	2040	2050
Eastern region	Current balance	0.30	0.10	−0.05	−0.18	−0.30
	Cumulative surplus	4.70	4.25	3.55	2.40	0.95
Central region	Current balance	0.12	0.02	−0.15	−0.30	−0.45
	Cumulative surplus	1.85	1.55	1.05	0.15	−0.90
Western region	Current balance	0.05	−0.05	−0.18	−0.35	−0.55
	Cumulative surplus	1.20	1.05	0.60	−0.20	−1.80

In terms of current balances, the eastern region’s surplus moderates somewhat under national pooling, yet the central and western regions experience substantial relief. Relative to the baseline, the 2050 current deficit narrows from −0.95 to −0.45 trillion yuan in the central region and from −1.20 to −0.55 trillion yuan in the western region, while the eastern region’s 2050 current balance improves from −0.62 to −0.30 trillion yuan.

With respect to cumulative balances, the national pooling scenario significantly postpones the point at which the central and western regions’ reserves turn negative and reduces the magnitude of long-run deficits. By 2050, cumulative balances equal 0.95 trillion yuan in the eastern region, −0.90 trillion yuan in the central region, and −1.80 trillion yuan in the western region, all materially stronger than the corresponding baseline outcomes. The effect of pooling should therefore be interpreted as a compositional redistribution of fiscal pressure across provinces rather than as an autonomous expansion of national pension revenues.

#### 3.3.3 Synergy scenario: national pooling with delayed retirement

When national pooling and delayed retirement are implemented jointly, the financial position of the pension insurance fund improves further relative to either single-policy scenario, as shown in Table 4.

**Table 4:** Policy synergy scenario (unit: trillion yuan).

Region	Indicators	2025	2030	2035	2040	2050
Eastern region	Current balance	0.45	0.30	0.10	−0.05	−0.15
	Cumulative surplus	4.90	4.75	4.30	3.55	2.10
Central region	Current balance	0.20	0.12	0.00	−0.12	−0.28
	Cumulative surplus	2.05	1.95	1.70	1.10	0.05
Western region	Current balance	0.10	0.05	−0.05	−0.15	−0.30
	Cumulative surplus	1.40	1.35	1.15	0.70	−0.20

Current balances across all three regions under the combined scenario exceed those observed in either the delayed-retirement-only or the national-pooling-only scenario. By 2050, current balances improve to −0.15, −0.28, and −0.30 trillion yuan in the eastern, central, and western regions, compared with scenario under pooling only and delayed retirement only.

Regarding cumulative balances, the combined scenario yields 2050 reserves of 2.10 trillion yuan in the eastern region, 0.05 trillion yuan in the central region, and −0.20 trillion yuan in the western region, outperforming both stand-alone reforms. The interaction term, defined as combined minus pooling-only minus delayed-retirement-only plus baseline, is positive in every benchmark year. By 2050, the cumulative interaction effect reaches 8.97 trillion yuan in the eastern region, 8.70 trillion yuan in the central region, and 12.74 trillion yuan in the western region, confirming that the joint reform package performs better than the arithmetic sum of the two separate reforms.

### 3.4 Robustness, province-level heterogeneity, and back-testing evidence

Table 5 reports a set of sensitivity tests designed to respond directly to the reviewer’s concern about uncertainty. The informal 10% and informal 20% scenarios show that weaker compliance among workers in the extended retirement-age brackets further reduces the already fragile stand-alone delayed-retirement results, while the pessimistic macro scenario produces the most severe deterioration in all three regions. Alternative assumptions for higher pension indexation, lower collection rates, and lower fund returns also weaken the cumulative reserve path, whereas the low-fertility variant exerts a comparatively limited effect in the near term but becomes more visible by 2050. In implementation terms, the Informal 10% and Informal 20% cases scale down only the incremental contribution inflow generated by workers who remain in formal insured employment because of delayed retirement; they do not mechanically enlarge the retiree pool or alter the benefit formula within the same period. This is why the quantitative effect, while consistently negative, remains relatively muted: the shock applies to a narrow margin of additional older contributors rather than to the full stock of insured workers, so even a 20% compliance loss among the affected group translates into a smaller aggregate deterioration than a 20% loss applied to the entire contribution base. From a policy perspective, the results in Table 5 indicates that sustained macroeconomic weakness poses the greatest threat to the projected reform gains, while weaker collection and lower fund returns represent the next most important risks.

**Table 5:** Sensitivity analysis (unit: trillion yuan).

Scenario	Year	Eastern region		Central region		Western region	
		Current	Cumulative	Current	Cumulative	Current	Cumulative
Informal 10%	2025	0.4	4.84	0.02	1.56	−0.12	0.93
	2030	−0.01	3.74	−0.32	0.41	−0.55	−0.48
	2035	−0.24	1.51	−0.63	−1.78	−0.88	−3.65
	2040	−0.28	−0.91	−0.81	−4.54	−1.09	−7.82
	2050	−1.36	−8.96	−1.34	−11.86	−1.71	−18.66
Informal 20%	2025	0.4	4.84	0.02	1.56	−0.12	0.93
	2030	−0.01	3.73	−0.32	0.41	−0.55	−0.48
	2035	−0.25	1.48	−0.63	−1.78	−0.88	−3.65
	2040	−0.3	−1.03	−0.81	−4.55	−1.09	−7.83
	2050	−1.36	−9.3	−1.34	−11.86	−1.71	−18.67
Pessimistic macro	2025	0.32	4.64	0	1.51	−0.15	0.86
	2030	−0.15	2.18	−0.36	0.13	−0.6	−0.86
	2035	−0.52	−2.66	−0.72	−2.28	−0.99	−4.37
	2040	−0.77	−9.38	−0.99	−5.21	−1.29	−8.82
	2050	−2.37	−31.49	−1.73	−11.84	−2.18	−18.96
Low fertility	2025	0.42	4.85	0.05	1.60	−0.10	0.95
	2030	0.18	4.40	−0.18	0.95	−0.35	0.20
	2035	−0.12	3.20	−0.45	−0.20	−0.62	−1.50
	2040	−0.35	1.35	−0.72	−1.80	−0.88	−3.80
	2050	−0.67	−0.96	−0.97	−4.16	−1.22	−7.58
High pension indexation	2025	0.42	4.85	0.05	1.60	−0.10	0.95
	2030	0.16	4.35	−0.19	0.93	−0.36	0.17
	2035	−0.20	2.86	−0.48	−0.35	−0.66	−1.68
	2040	−0.55	0.13	−0.81	−2.34	−0.98	−4.44
	2050	−1.34	−8.19	−1.25	−7.27	−1.57	−11.34
Lower collection rate	2025	0.21	4.64	−0.02	1.53	−0.18	0.87
	2030	−0.06	2.88	−0.26	0.46	−0.45	−0.40
	2035	−0.39	−0.14	−0.54	−1.31	−0.73	−2.84
	2040	−0.64	−4.45	−0.82	−3.75	−1.00	−6.16
	2050	−0.90	−13.86	−1.06	−8.63	−1.33	−12.95
Lower fund return	2025	0.42	4.73	0.05	1.58	−0.10	0.91
	2030	0.18	3.52	−0.18	0.88	−0.35	0.07
	2035	−0.12	0.85	−0.45	−0.17	−0.62	−1.59
	2040	−0.35	−3.13	−0.72	−1.32	−0.88	−3.49
	2050	−0.62	−10.09	−0.95	−0.32	−1.20	−3.63

In the sensitivity analysis, Informal 10%, Informal 20%, Pessimistic macro, Low Fertility, High Pension Indexation, Lower Collection Rate, and Lower Fund Return vary one parameter block at a time relative to the anchored baseline benchmark. Informal 10%: a 10% compliance loss among workers in the extended retirement-age brackets. Informal 20%: a 20% compliance loss among workers in the extended retirement-age brackets. Pessimistic macro: (a) structurally lower long-run wage growth, (b) weaker effective contribution collection, and (c) a lower fund investment return. More specifically, Informal 10% and Informal 20% are implemented by reducing only the incremental contribution revenue generated by workers retained in formal insured employment because of delayed retirement by 10 and 20%, respectively, while leaving retirement timing and the benefit formula unchanged. The Low-Fertility scenario increases the annual birth-decline parameter from 0 to 1%, so that newborn cohorts evolve according to (1 − Birth_decline)^(year − 2020). The High-Pension-Indexation scenario raises the long-run pension growth rate from 2.6 to 3.2%. The Lower-Collection-Rate scenario scales contribution revenue by 0.95, which is equivalent to reducing the effective collection rate from 0.93 to 0.8835. The Lower-Fund-Return scenario reduces the fund investment return from 5 to 3%. The Pessimistic-Macroeconomic scenario is implemented on top of the delayed-retirement scenario and assumes lower long-run wage growth, from 3.4 to 2.0%, weaker effective contribution collection, with revenue scaled by 0.98, and a lower fund return, from 5 to 3%, while keeping the long-run pension growth rate at 2.6%.

Tables 6, 7 provide the province-level evidence that regional averages conceal substantial heterogeneity. Under the baseline scenario, Guangdong still records a cumulative surplus of 41.28 trillion yuan in 2050 and Fujian 10.21 trillion yuan, whereas Shanghai falls to −22.90 trillion yuan, Hubei to −17.75 trillion yuan, and Gansu to −6.41 trillion yuan. The national total reported in Table 6 turns from a positive cumulative balance of 10.54 trillion yuan in 2035 to −51.32 trillion yuan in 2050, while Table 7 shows that the within-region dispersion is especially large in the eastern and western regions.

**Table 6:** Province-level and national-level baseline (unit: trillion yuan).

Province	Region	Year	Current balance	Cumulative surplus
Beijing	Eastern region	2025	0.18	0.94
Beijing		2030	0.19	2.28
Beijing		2035	0.13	3.79
Beijing		2040	0.03	5.26
Beijing		2050	−0.32	6.85
Tianjin		2025	−0.02	−0.02
Tianjin		2030	−0.05	−0.24
Tianjin		2035	−0.09	−0.73
Tianjin		2040	−0.15	−1.62
Tianjin		2050	−0.28	−5.31
Hebei		2025	−0.03	−0.02
Hebei		2030	−0.08	−0.37
Hebei		2035	−0.13	−1.06
Hebei		2040	−0.20	−2.27
Hebei		2050	−0.36	−7.15
Shanghai		2025	−0.07	0.01
Shanghai		2030	−0.21	−0.83
Shanghai		2035	−0.41	−2.86
Shanghai		2040	−0.66	−6.70
Shanghai		2050	−1.24	−22.90
Jiangsu		2025	0.05	0.46
Jiangsu		2030	−0.04	0.57
Jiangsu		2035	−0.11	0.29
Jiangsu		2040	−0.23	−0.62
Jiangsu		2050	−0.52	−5.60
Zhejiang		2025	0.07	0.24
Zhejiang		2030	−0.02	0.41
Zhejiang		2035	−0.15	0.00
Zhejiang		2040	−0.32	−1.36
Zhejiang		2050	−0.76	−8.90
Fujian		2025	0.17	0.42
Fujian		2030	0.19	1.53
Fujian		2035	0.20	3.01
Fujian		2040	0.19	4.92
Fujian		2050	0.14	10.21
Shandong		2025	0.03	0.17
Shandong		2030	−0.03	0.18
Shandong		2035	−0.09	−0.14
Shandong		2040	−0.17	−0.92
Shandong		2050	−0.31	−4.29
Guangdong		2025	0.64	2.45
Guangdong		2030	0.72	6.91
Guangdong		2035	0.76	12.91
Guangdong		2040	0.75	20.66
Guangdong		2050	0.41	41.28
Hainan		2025	0.01	0.06
Hainan		2030	0.00	0.12
Hainan		2035	−0.01	0.15
Hainan		2040	−0.02	0.11
Hainan		2050	−0.06	−0.34
Shanxi	Central region	2025	−0.05	0.01
Shanxi		2030	−0.07	−0.31
Shanxi		2035	−0.10	−0.86
Shanxi		2040	−0.13	−1.75
Shanxi		2050	−0.24	−5.17
Anhui		2025	0.03	0.25
Anhui		2030	0.00	0.39
Anhui		2035	−0.03	0.42
Anhui		2040	−0.06	0.27
Anhui		2050	−0.14	−0.81
Jiangxi		2025	0.00	0.06
Jiangxi		2030	−0.04	−0.05
Jiangxi		2035	−0.08	−0.39
Jiangxi		2040	−0.12	−1.05
Jiangxi		2050	−0.20	−3.73
Henan		2025	0.06	0.23
Henan		2030	0.05	0.57
Henan		2035	0.06	1.03
Henan		2040	0.03	1.57
Henan		2050	−0.05	2.61
Hubei		2025	−0.10	−0.11
Hubei		2030	−0.22	−1.08
Hubei		2035	−0.34	−2.98
Hubei		2040	−0.49	−6.15
Hubei		2050	−0.73	−17.75
Hunan		2025	0.01	0.15
Hunan		2030	−0.01	0.20
Hunan		2035	−0.02	0.18
Hunan		2040	−0.04	0.05
Hunan		2050	−0.11	−0.81
Neimenggu	Western region	2025	−0.05	−0.07
Neimenggu		2030	−0.08	−0.44
Neimenggu		2035	−0.11	−1.10
Neimenggu		2040	−0.15	−2.13
Neimenggu		2050	−0.23	−5.80
Guangxi		2025	−0.01	0.03
Guangxi		2030	−0.04	−0.12
Guangxi		2035	−0.07	−0.48
Guangxi		2040	−0.10	−1.10
Guangxi		2050	−0.18	−3.55
Chongqing		2025	0.00	0.11
Chongqing		2030	−0.02	0.10
Chongqing		2035	−0.05	−0.07
Chongqing		2040	−0.07	−0.42
Chongqing		2050	−0.15	−2.02
Sichuan		2025	0.05	0.37
Sichuan		2030	0.02	0.64
Sichuan		2035	−0.01	0.84
Sichuan		2040	−0.04	0.92
Sichuan		2050	−0.18	0.34
Guizhou		2025	0.02	0.14
Guizhou		2030	0.02	0.28
Guizhou		2035	0.01	0.42
Guizhou		2040	0.00	0.54
Guizhou		2050	−0.03	0.72
Yunnan		2025	0.00	0.13
Yunnan		2030	−0.05	0.01
Yunnan		2035	−0.11	−0.44
Yunnan		2040	−0.17	−1.37
Yunnan		2050	−0.30	−5.21
Tibet		2025	0.00	0.02
Tibet		2030	0.00	0.02
Tibet		2035	−0.01	0.01
Tibet		2040	−0.01	−0.04
Tibet		2050	−0.04	−0.38
Shaanxi		2025	0.03	0.15
Shaanxi		2030	0.02	0.30
Shaanxi		2035	0.00	0.42
Shaanxi		2040	−0.04	0.42
Shaanxi		2050	−0.15	−0.39
Gansu		2025	−0.04	−0.05
Gansu		2030	−0.08	−0.41
Gansu		2035	−0.13	−1.12
Gansu		2040	−0.17	−2.27
Gansu		2050	−0.25	−6.41
Qinghai		2025	−0.01	−0.01
Qinghai		2030	−0.02	−0.10
Qinghai		2035	−0.03	−0.26
Qinghai		2040	−0.04	−0.52
Qinghai		2050	−0.07	−1.54
Ningxia		2025	0.01	0.03
Ningxia		2030	0.00	0.07
Ningxia		2035	0.00	0.10
Ningxia		2040	−0.01	0.10
Ningxia		2050	−0.04	−0.11
Xinjiang		2025	−0.02	0.09
Xinjiang		2030	−0.05	−0.09
Xinjiang		2035	−0.09	−0.54
Xinjiang		2040	−0.15	−1.36
Xinjiang		2050	−0.33	−5.18
	National level	2025	0.95	6.24
		2030	0.10	10.56
		2035	−1.01	10.54
		2040	−2.55	3.17
		2050	−6.70	−51.32

**Table 7:** Decomposition of regional differences (unit: trillion yuan).

Region	Province	Current	Cumulative	Current	Cumulative
		2035		2050	
Eastern region	Shanghai	−0.41	−2.86	−1.24	−22.90
	Zhejiang	−0.15	0.00	−0.76	−8.90
	Hebei	−0.13	−1.06	−0.36	−7.15
	Jiangsu	−0.11	0.29	−0.52	−5.60
	Tianjin	−0.09	−0.73	−0.28	−5.31
	Shandong	−0.09	−0.14	−0.31	−4.29
	Hainan	−0.01	0.15	−0.06	−0.34
	Beijing	0.13	3.79	−0.32	6.85
	Fujian	0.20	3.01	0.14	10.21
	Guangdong	0.76	12.91	0.41	41.28
Central region	Hubei	−0.34	−2.98	−0.73	−17.75
	Shanxi	−0.10	−0.86	−0.24	−5.17
	Jiangxi	−0.08	−0.39	−0.20	−3.73
	Anhui	−0.03	0.42	−0.14	−0.81
	Hunan	−0.02	0.18	−0.11	−0.81
	Henan	0.06	1.03	−0.05	2.61
Western region	Gansu	−0.13	−1.12	−0.25	−6.41
	Neimenggu	−0.11	−1.10	−0.23	−5.80
	Yunnan	−0.11	−0.44	−0.30	−5.21
	Xinjiang	−0.09	−0.54	−0.33	−5.18
	Guangxi	−0.07	−0.48	−0.18	−3.55
	Chongqing	−0.05	−0.07	−0.15	−2.02
	Qinghai	−0.03	−0.26	−0.07	−1.54
	Sichuan	−0.01	0.84	−0.18	0.34
	Tibet	−0.01	0.01	−0.04	−0.38
	Shaanxi	0.00	0.42	−0.15	−0.39
	Ningxia	0.00	0.10	−0.04	−0.11
	Guizhou	0.01	0.42	−0.03	0.72

Table 8 reports the provincial calibration and back-testing panel for 2020–2023, including workers, retirees, wages, pension levels, revenue, expenditure, and cumulative balances. These observed provincial series are used to anchor the initial conditions of the simulation and to ensure that the projection starts from the actual operational structure of the pension insurance fund rather than from stylized national averages alone.

**Table 8:** Provincial-level back-testing.

Region	Year	Province	Workers	Retirees	Wage	Pension	Revenue	Expenditure	Cumulative
Eastern region	2020	Beijing	1,466	311	112,886	62,726	2,161	1953	5,763
		Tianjin	497	234	81,324	45,273	855	1,061	359
		Hebei	1,258	480	64,910	41,275	1,684	1982	642
		Shanghai	1,095	522	124,056	57,156	2037	2,982	1,214
		Jiangsu	2,593	965	82,344	36,977	3,018	3,566	4,232
		Zhejiang	2,314	897	79,133	38,070	2,397	3,416	2,458
		Fujian	992	209	73,517	42,502	791	888	710
		Shandong	2,292	754	74,906	41,932	2,491	3,162	1,476
		Guangdong	4,162	711	91,764	46,598	3,858	3,314	12,338
		1Hainan	231	74	78,516	40,417	266	300	249
	2021	Beijing	1,508	319	127,535	65,041	3,266	2075	6,517
		Tianjin	527	238	89,736	50,273	1,160	1,196	327
		Hebei	1,314	492	69,465	43,567	2076	2,142	624
		Shanghai	1,126	528	136,757	60,977	3,380	3,222	1,225
		Jiangsu	2,608	1,002	88,543	39,994	4,391	4,007	4,414
		Zhejiang	2,456	912	89,240	40,983	3,489	3,737	2,128
		Fujian	1,110	220	79,848	42,222	1,065	927	707
		Shandong	2,438	789	79,597	44,147	3,454	3,483	1,386
		Guangdong	4,327	752	99,720	46,304	6,113	3,484	14,110
		Hainan	253	76	89,832	41,748	388	318	319
	2022	Beijing	1,540	328	135,567	71,353	3,436	2,342	7,286
		Tianjin	556	244	95,028	51,622	1,225	1,258	355
		Hebei	1,364	503	74,533	46,094	2,294	2,320	615
		Shanghai	1,124	536	146,196	64,795	3,706	3,470	1,428
		Jiangsu	2,639	1,051	95,231	40,010	4,847	4,206	4,878
		Zhejiang	2,525	948	96,237	41,710	3,797	3,952	1883
		Fujian	1,442	231	84,354	43,855	1,221	1,015	832
		Shandong	2,511	823	84,831	44,520	3,559	3,662	1,321
		Guangdong	4,432	797	105,684	45,767	6,420	3,649	15,723
		Hainan	274	79	83,356	42,170	429	332	382
	2023	Beijing	1,567	338	141,133	70,394	4,009	2,378	8,614
		Tianjin	568	250	100,260	51,631	1,311	1,288	442
		Hebei	1,429	521	78,411	46,619	2,435	2,428	620
		Shanghai	1,147	543	147,684	67,797	4,226	3,680	1951
		Jiangsu	2,646	1,106	98,292	40,735	5,183	4,505	5,341
		Zhejiang	2,584	990	99,722	43,212	3,908	4,276	1,381
		Fujian	1,544	245	88,976	44,615	1,367	1,094	1,006
		Shandong	2,560	864	88,313	45,873	3,995	3,965	1,345
		Guangdong	4,518	851	110,004	46,434	7,037	3,950	17,549
		Hainan	292	82	89,298	44,357	499	362	485
Central region	2020	Shanxi	655	278	64,708	46,391	1,112	1,291	1,505
		Anhui	914	369	71,702	36,960	1,314	1,364	1863
		Jiangxi	807	361	66,576	32,515	1,033	1,173	724
		Henan	1724	524	63,936	37,185	1,690	1950	1,079
		Hubei	1,148	597	71,110	37,961	2025	2,266	963
		Hunan	1,222	503	65,520	34,440	1734	1731	1865
	2021	Shanxi	711	292	70,968	47,995	1,429	1,401	1,596
		Anhui	1,003	381	76,643	38,572	1732	1,470	2,130
		Jiangxi	876	371	70,560	34,367	1,334	1,276	843
		Henan	1841	536	68,172	39,457	2,137	2,115	1,131
		Hubei	1,218	617	77,425	39,569	2,442	2,442	1,104
		Hunan	1,328	522	71,724	35,918	1860	1874	1864
	2022	Shanxi	751	315	77,257	47,058	1,413	1,481	1,525
		Anhui	1,183	398	85,682	38,897	1884	1,548	2,349
		Jiangxi	977	385	73,176	35,814	1,390	1,379	878
		Henan	1920	565	71,580	38,828	2,292	2,194	1,310
		Hubei	1,319	641	82,196	42,295	2,550	2,711	959
		Hunan	1,352	541	75,408	37,061	2020	2004	1863
	2023	Shanxi	778	324	82,255	50,895	1,681	1,650	1,552
		Anhui	1,271	417	84,533	41,162	2,174	1715	2,692
		Jiangxi	1,028	408	76,764	36,151	1,523	1,475	925
		Henan	1988	591	75,120	40,080	2,566	2,367	1,591
		Hubei	1,379	669	85,621	44,745	3,159	2,993	1,135
		Hunan	1,454	565	80,532	38,105	2,210	2,151	1895
Western region	2020	Neimenggu	475	311	76,128	40,835	997	1,271	408
		Guangxi	645	275	69,832	40,710	983	1,118	631
		Chongqing	782	422	73,978	30,697	1,213	1,295	1,026
		Sichuan	1883	948	74,520	32,770	2,662	3,105	3,367
		Guizhou	554	161	76,547	42,598	668	684	878
		Yunnan	516	186	75,408	43,351	820	805	1,341
		Tibet	42	10	106,068	113,654	132	118	185
		Shanxi	885	273	72,636	46,970	1,222	1,282	748
		Gansu	320	165	72,756	40,759	559	671	377
		Qinghai	110	49	84,278	57,629	238	280	23
		Ningxia	172	69	73,320	42,128	265	289	242
		Xinjiang	543	224	78,372	48,315	1,088	1,084	1,330
	2021	Neimenggu	503	320	81,012	43,119	1,195	1,380	336
		Guangxi	704	281	74,362	42,529	1,278	1,196	718
		Chongqing	912	443	79,133	32,530	1738	1,440	1,335
		Sichuan	2,202	977	81,420	34,246	3,597	3,346	3,717
		Guizhou	591	165	81,570	43,153	864	712	1,031
		Yunnan	551	189	79,464	47,240	1,077	892	1,556
		Tibet	49	11	118,800	117,064	160	128	217
		Shanxi	947	283	78,520	53,401	1,605	1,509	851
		Gansu	334	169	76,800	43,525	694	733	378
		Qinghai	119	50	91,253	58,220	272	291	26
		Ningxia	182	70	85,252	44,631	302	314	231
		Xinjiang	562	230	85,066	51,562	1,337	1,185	1,494
	2022	Neimenggu	560	335	89,630	43,367	1,248	1,454	350
		Guangxi	746	287	77,268	42,638	1,313	1,222	769
		Chongqing	980	452	82,348	33,113	1,688	1,496	1,507
		Sichuan	2,321	1,007	84,912	34,185	3,702	3,441	3,881
		Guizhou	599	171	82,291	43,785	929	750	1,145
		Yunnan	611	196	82,872	47,933	1,154	939	1,689
		Tibet	51	11	129,492	119,730	172	133	239
		Shanxi	992	294	84,344	50,630	1,696	1,487	1,002
		Gansu	343	175	81,792	45,427	718	793	343
		Qinghai	124	52	96,348	61,272	291	318	22
		Ningxia	211	73	91,993	45,328	348	332	247
		Xinjiang	582	235	91,501	52,060	1,408	1,226	1,624
	2023	Neimenggu	585	347	97,260	44,306	1,422	1,538	481
		Guangxi	775	294	81,070	44,676	1,468	1,315	874
		Chongqing	1,004	471	87,169	33,865	1,639	1,596	1,512
		Sichuan	2,379	1,047	90,220	35,410	4,050	3,708	4,101
		Guizhou	617	177	87,267	45,186	1,014	802	1,285
		Yunnan	681	203	86,124	50,656	1,246	1,026	1822
		Tibet	56	11	138,552	126,579	215	144	291
		Shanxi	1,044	302	91,180	52,080	1911	1,575	1,282
		Gansu	352	180	86,328	48,329	845	870	350
		Qinghai	134	54	103,711	62,528	337	339	44
		Ningxia	216	76	97,054	47,536	391	361	275
		Xinjiang	622	246	99,984	54,043	1,567	1,330	1815

Workers and retirees are measured in 10,000 yuan; wage and pension are in yuan; revenue, expenditure, and cumulative are in billion yuan.

## 4 Analysis and discussion

### 4.1 Operational characteristics of the pension fund across policy scenarios

The numerical simulation results presented in Section 3 reveal substantial variation in the operational trajectories of China’s urban employee basic pension insurance fund across policy scenarios. These differences are evident not only in the magnitude of fund balances but also in the evolution of current revenue–expenditure pressures and the structural configuration of regional risk exposure.

Under the baseline scenario, pension fund dynamics exhibit a pronounced pattern of intensifying regional divergence. The eastern region, benefiting from a comparatively high level of economic development, a stable employment base, and relatively ample historical fund accumulation, maintains positive current and cumulative surpluses in the early years of the projection. However, as population aging deepens and the retiree population expands, its current surplus steadily erodes and the safety margin of its cumulative reserves contracts. In contrast, the central and western regions are constrained by net labor outflows, elevated dependency ratios, and insufficient historical accumulation, causing current revenue–expenditure pressure to materialize earlier and evolve into persistent deficits by the middle of the projection horizon. This divergence pattern is broadly consistent with the findings of Zhao et al., who project that the national fund enters deficit in 2027 and that less developed provinces face substantially earlier depletion dates (16). Our provincial level projections refine this picture: the western region’s cumulative balance turns negative as early as 2030, roughly a decade ahead of the central region (2035) and two decades ahead of the eastern region (2050). This temporal gradient that west before center before east, is a direct consequence of the interaction between demographic aging trajectories and provincial economic fundamentals. The magnitude of the east–west gap we document (approximately 20 years in depletion timing) is larger than reported in Chen and Shang who project a national insolvency point near 2050 under similar baseline assumptions, underscoring the risk that aggregate analyses mask severe subnational fiscal stress (2).

The introduction of the national pooling mechanism alters the fund’s operational trajectory in important ways. Because the central adjustment fund is modeled as a balanced pay-in/pay-out system, the aggregate national current balance is unchanged by construction; what changes is the provincial distribution of surpluses and deficits. The simulation results indicate that national pooling substantially narrows the current revenue–expenditure gaps in the central and western regions and meaningfully postpones the point at which their cumulative reserves become negative. Although the eastern region experiences a degree of reserve transfer under the pooling arrangement, its long-run cumulative balance remains relatively stable, and no systemic fiscal imbalance emerges. These findings provide quantitative validation of the institutional value of national pooling in attenuating regional disparities and dispersing localized risks. They demonstrate that cross regional fiscal transfers can substantially improve the interprovincial balance of pension fund operations without altering the underlying institutional parameters.

Under the coordinated implementation of national pooling and delayed retirement, the fund’s operational position improves further. The four-scenario comparison shows that the combined reform outperforms both stand-alone policies and generates a positive interaction effect, especially in cumulative balances after 2035. This pattern suggests that individual policy instruments generate diminishing marginal returns when deployed in isolation, whereas institutional coordination amplifies the overall stability of fund operations to a considerably greater degree.

### 4.2 Mechanisms and limitations of the national pooling policy

From a mechanistic standpoint, the national pooling system achieves spatial risk sharing by establishing cross regional channels for fund transfers, reallocating resources from provinces with relatively ample surpluses to those confronting significant deficits. The core function of this mechanism is to narrow disparities in payment capacity across provinces and mitigate the probability of systemic stress triggered by adverse demographic dynamics in specific localities.

The simulation results indicate that national pooling yields a particularly pronounced short-run improvement in current balances, exerting a notable stabilizing effect on the central and western regions. Its effect arises through redistribution rather than through a change in aggregate contributions, aggregate benefits, or aggregate investment returns. This distinction is important for interpreting Table 3 and for understanding why pooling alone cannot eliminate the secular pressure created by aging. This interpretation aligns with Chen and Shang, who demonstrate that national pooling, even when combined with a reduced 16% contribution rate and collection responsibility transferred to taxation authorities, can postpone insolvency to the distant future, but does so through improved collection efficiency rather than through the redistributive channel per se (2). Our results suggest a more cautious assessment: while pooling materially narrows current deficits in recipient provinces, the long-run cumulative balance trajectories remain on declining paths in all three regions, implying that the redistributive mechanism alone cannot fully offset the secular pressure of population aging.

Furthermore, the national pooling system may encounter incentive and commitment problems over its long-run operation. Provinces with weaker administrative capacity may also struggle to enforce contribution compliance after retirement-age reform. This concern is especially relevant for some western provinces, where the gains projected under full implementation could be overstated if tax and social-insurance collection remain comparatively weak.

### 4.3 The impact of delayed retirement on fund revenue and expenditure

The delayed retirement policy influences both the revenue and expenditure sides of the pension insurance fund by altering the retirement behavior of workers. On the revenue side, postponing retirement expands the pool of employed contributors and extends contribution durations, thereby augmenting fund income and boosting individual account accumulations. On the expenditure side, it directly reduces the inflow of new retirees in each period, deferring the concentrated release of pension obligations. This dual effect that expenditure containment coupled with revenue enhancement, renders delayed retirement a uniquely potent instrument for improving the intertemporal balance of the pension insurance fund.

The numerical simulation results indicate that the beneficial effects of delayed retirement materialize gradually and are most pronounced over the medium to long term. The improvements in current and cumulative balances under this policy substantially exceed those achieved through interregional fiscal transfers alone. This lagged response is fully consistent with the phased implementation schedule of the reform, underscoring that delayed retirement is not an immediate remedy but rather a structural adjustment whose impact compounds over time. Our finding that delayed retirement delivers larger long-run improvements than spatial redistribution is consistent with Wu et al., who show in an overlapping generations framework that even a voluntary delayed retirement scheme can substantially improve fund sustainability relative to parametric adjustments alone (15). However, their analysis suggests that voluntary schemes dominate mandatory ones in welfare terms—a dimension our macro actuarial framework cannot evaluate. Wang similarly finds that combining retirement age postponement with contribution rate adjustments achieves a more favorable adequacy–sustainability trade-off than either instrument alone (14). Our results extend these findings by showing that the temporal channel and the spatial channel are complementary rather than substitutable: the former improves the aggregate fiscal position, while the latter ensures that the improvement is distributed across provinces in a manner that compresses, rather than widens, regional disparities.

Nevertheless, the delayed retirement policy confronts important practical constraints. The model does not separately endogenize flexible retirement choices, occupational differences, health heterogeneity, or employer-side retention responses. Table 5 therefore provides an explicit sensitivity check for two informal-sector compliance losses among workers in the extended retirement-age brackets, alongside alternative macro, collection, return, fertility, and pension-indexation assumptions.

### 4.4 Synergistic effects and practical implications

By incorporating national pooling and delayed retirement into a unified analytical framework, we obtain a more rigorous identification of their joint contribution to pension sustainability. The interaction term is computed as combined scenario minus pooling-only scenario minus delayed-retirement-only scenario plus baseline, and it is positive for both current and cumulative balances across the eastern, central, and western regions. National pooling operates primarily along the spatial dimension, attenuating regional disparities through cross provincial fiscal transfers. Delayed retirement, by contrast, operates along the temporal dimension, improving the intertemporal balance of the fund by extending contribution periods and deferring the release of benefit expenditures. When the two policies are implemented jointly, the spatial risk-sharing mechanism and the temporal adjustment mechanism reinforce one another, generating a marked improvement in the overall stability of the pension insurance system.

The simulation results confirm that under the combined scenario, not only do current balances improve across all three regions, but the long-run trajectory of cumulative balances also becomes more stable. The interaction effect is especially pronounced in the cumulative balance by 2050, reaching 8.97 trillion yuan in the east, 8.70 trillion yuan in the center, and 12.74 trillion yuan in the west. These magnitudes indicate that policy coordination changes the dynamic path of reserves rather than merely adding two independent one-period effects.

It is important to emphasize that the effectiveness of coordinated policies depends critically on the refinement of institutional design. On the one hand, the rules governing national pooling and fiscal transfers must be further clarified and rendered more transparent to prevent imbalances in the distribution of responsibilities across provinces. On the other hand, delayed retirement should be advanced together with employment support for older workers, occupational health protection, and differentiated enforcement arrangements across provinces. In regions with limited administrative capacity, especially parts of western China, the projected gains could otherwise be overstated.

### 4.5 Boundary conditions and external validity of policy conclusions

The policy conclusions drawn from the preceding analysis are conditional on the modeling framework, parameter assumptions, and scenario design employed in this study. It is therefore essential to delineate the boundary conditions under which our findings should be interpreted and the respects in which extrapolation beyond these conditions may be unwarranted.

First, the demographic projections are conditioned on the provincial fertility and mortality patterns observed in the 2020 Census and the China Resident Life Table. Should China’s total fertility rate deviate substantially from the assumed range (approximately 1.0–1.3)—whether through a sustained decline below 1.0 or a policy induced recovery—the long-run size and age structure of the contributor base would differ materially from our projections. Similarly, continued improvements in old age mortality beyond those embedded in the current life tables would increase the duration of benefit receipt and amplify fund expenditure relative to our baseline. While the directional effect of these demographic uncertainties is clear, the precise magnitude is sensitive to assumptions that will require updating as new census and survey data become available.

Second, the simulations adopt province specific wage growth trajectories that are calibrated to historical averages and are assumed to converge gradually toward a lower long-run growth path. The sensitivity analysis in Table 5 shows that pessimistic macro conditions produce the sharpest deterioration among the alternative cases. For example, by 2050 the eastern region’s cumulative balance falls to −31.49 trillion yuan under the pessimistic macro scenario, far below the corresponding outcomes under the informal-sector and lower-return variants. This confirms that the main conclusions are highly sensitive to sustained macroeconomic weakness. Taken together, these sensitivity comparisons imply that preserving the gains from coordinated pension reform depends less on marginal short-run demographic variation than on sustaining wage growth, contribution compliance, and investment performance. Policy coordination should therefore prioritize employment stabilization, stricter collection governance, and prudent reserve-management arrangements, especially once delayed retirement begins to rely more heavily on continued formal-sector participation.

Third, the policy simulations assume full and uniform implementation of the legislated reforms. In practice, the phased rollout of delayed retirement may encounter variation in enforcement intensity across provinces, industries, and enterprise ownership types. Prior experience with social insurance compliance in China (2) suggests that effective collection rates and retirement age enforcement are likely to be weaker in provinces with larger informal sectors and more limited administrative capacity. This caution is particularly relevant for the western region, where the residual deficit risk persists even under the full-implementation combined scenario and where Table 5 shows that compliance losses further weaken the projected gains.

Fourth, the macro actuarial framework does not endogenize individual behavioral responses to policy changes. Workers may respond to delayed retirement by altering labor supply at the intensive margin, adjusting savings behavior, or shifting into informal employment to avoid extended contribution requirements (13). Employers may respond by adjusting hiring and retention practices for older workers. These behavioral margins, which are not captured in our aggregate projection framework, could either amplify or attenuate the simulated policy effects. In particular, if delayed retirement induces a nontrivial shift toward informal employment among older workers nearing the original statutory retirement age, the effective expansion of the contribution base would be smaller than the mechanical simulation implies, and the improvement in fund sustainability would be correspondingly weaker. Integrating micro level behavioral estimates into the provincial projection framework represents an important direction for future work.

In summary, the quantitative projections presented in this paper are best interpreted as conditional forecasts that map a specific set of demographic, economic, and policy assumptions into fund-balance trajectories, rather than as unconditional predictions of the future state of China’s pension system. The qualitative findings that national pooling redistributes provincial fiscal pressure, that delayed retirement alone is insufficient in the present calibration, and that the joint implementation of the two reforms generates a positive interaction effect are robust to a range of alternative assumptions. The precise numerical trajectories, however, should be updated as new demographic data, economic forecasts, and policy implementation experience accumulate.

## 5 Conclusion

First, under a counterfactual of unchanged policies, China’s urban employee basic pension insurance fund confronts pronounced regionally differentiated sustainability risks. The baseline simulation shows that the western region enters cumulative deficit first, followed by the central region, while the eastern region preserves a longer reserve buffer but still turns negative by 2050. The province-level results further reveal large within-region dispersion, indicating that regional averages mask substantial subnational stress.

Second, the introduction of the national pooling mechanism significantly attenuates cross regional imbalances in fund operations. Its principal function is redistributive: by construction, it changes the provincial allocation of surpluses and deficits without creating new national aggregate resources. The policy therefore improves the fiscal position of recipient provinces, but it does not on its own reverse the long-run downward pressure generated by aging.

Third, the delayed-retirement-only scenario does not generate a stand-alone fiscal improvement in the present calibration. Relative to the baseline, isolated retirement postponement produces weaker current and cumulative balances by the end of the projection horizon, implying that the policy’s effectiveness depends on the broader institutional environment, implementation quality, and complementary adjustments.

Fourth, the pension fund performs optimally when national pooling and delayed retirement are implemented simultaneously. The combined scenario dominates both stand-alone reforms and produces a positive interaction term for both current and cumulative balances across all three regions, indicating that coordinated reform changes the medium- and long-run reserve trajectory in a genuinely non-additive way. Even under this coordinated policy configuration, however, certain regions continue to face residual shortfall risks by the end of the projection period.

In sum, the numerical simulation evidence presented in this paper indicates that national pooling can attenuate regional imbalances and that delayed retirement can substantially improve the medium- and long-run revenue–expenditure structure of the fund. The joint implementation of these two measures is conducive to strengthening the overall sustainability of China’s urban employee basic pension insurance fund, although it remains insufficient to fully eliminate the long-run fiscal pressures generated by population aging. Sensitivity analysis further shows that pessimistic macro conditions, weaker collection, and lower fund returns can materially erode these gains.

Drawing on the conclusions presented above, we argue that the reform of the pension insurance system should be pursued through coordinated multi-instrument design, regionally differentiated implementation, and dynamic adjustment. In particular, provinces with weaker administrative capacity should be supported through stronger collection governance, more transparent transfer rules, and complementary labor-market policies for older workers. Without these supporting conditions, the gains projected for deficit provinces, especially in the western region, may be difficult to realize in practice.

This study has several limitations that warrant acknowledgment. First, the macro actuarial framework does not capture heterogeneity in flexible retirement choices, occupational conditions, health status, or employer-side adjustment at the micro level. Second, the characterization of the national pooling mechanism is based on current policy rules and does not incorporate dynamic incentive effects or adaptive institutional responses. Third, the scenario projections remain conditional on assumed trajectories for population growth, wage growth, pension indexation, collection efficiency, and fund returns. These issues merit further investigation in subsequent research that integrates richer provincial data and more explicit behavioral mechanisms.

## Data Availability

The original contributions presented in the study are included in the article/Supplementary material, further inquiries can be directed to the corresponding author.
