# Supplementary material for: The impact of delayed retirement on the financial sustainability of China’s urban employee pension insurance fund
Source: Front Public Health. 2026 Aug 3;14:1895268. doi: 10.3389/fpubh.2026.1895268 (PMC13477983; doi:10.3389/fpubh.2026.1895268)
Supplement: Supplementary file 1 [file Data_Sheet_1.docx]

Supplementary Material

# 1 Supplementary Figures and Tables

## 1.1 Supplementary Figures

**Figure 1.** Historical Trend of National Pension Fund Surplus Changes.

**Figure 2.** Trend of Changes in National Pension Fund Income and Expenditure.

## 1.2 Supplementary Tables

Table A below documents the data sources, coverage, reference years, and key definitions used in the model calibration. All data were accessed through official public releases.

**Appendix Table A** Data source.

| **Data Category** | **Source** | **Reference Period** | **Coverage** | **Key Definitions / Notes** |
| --- | --- | --- | --- | --- |
| Population age structure | Seventh National Population Census (NBS) | 2020 | 31 provinces, full enumeration | Five-year age groups decomposed to single-year ages via proportional interpolation; serves as base-year population stock for cohort-component projection |
| Mortality rates | China Resident Life Table (National Health Commission) | 2020 (latest available) | National, by age and sex | Age-sex-specific mortality probabilities; applied in cohort-component method for natural attrition projection |
| Insured population (employed & retired) | MOHRSS Annual Statistical Bulletin; Provincial HMRSS operational reports | 2020–2023 | 31 provinces, urban employee pension scheme | Number of insured employed workers and retirees by province; used to calibrate coverage ratios and initial fund positions |
| Fund revenue & expenditure | MOHRSS Annual Statistical Bulletin; Provincial HMRSS operational reports | 2020–2023 | 31 provinces, urban employee pension scheme | Total fund revenue (contributions + fiscal subsidies), total expenditure (pension benefits), current surplus, cumulative balance |
| Average wage (urban units) | NBS Provincial Statistical Yearbooks | 2020–2023 | 31 provinces, urban non-private units | Average wage of employed persons in urban units; serves as contribution base and benefit calculation base proxy |
| Pension benefit adjustment rate | MOHRSS annual pension adjustment notices | 2020–2023 | National | Annual percentage increase in basic pension benefits; national average approx. 4.0–5.0% during this period |
| Collection rate | MOHRSS data; Chen & Shang (2021) | 2020–2023 | National average | Ratio of actual contributions collected to statutory contributions due; national average approx. 90.5%, with provincial variation |
| Fund investment return rate | National Council for Social Security Fund annual reports; provincial fund reports | 2020–2023 | National and provincial | Weighted average yield on pension fund assets (primarily bank deposits and government bonds); approx. 3.0–4.5% p.a. |
| Retirement age schedule (reform) | NPC Standing Committee Decision (Sept 2024) | 2025 onward | National, by worker category | Male: 60→63 (15-year phase-in); Female cadre: 55→58 (15-year phase-in); Female worker: 50→55 (15-year phase-in) |
| Minimum contribution period (reform) | State Council implementing measures (effective Jan 1, 2030) | 2030 onward | National | Increases from 15 to 20 years, phased in incrementally over 10 years at approximately 6 months per year |
| Central adjustment ratio | State Council Document No. 18 of 2018; implementation notices | 2022 onward | National | Started at 3.0% in 2022; scheduled to rise progressively; set to 3.5% from 2025 in simulations |
| Contribution rates | Comprehensive Plan for Reducing Social Insurance Premium Rates (2019) | 2019 onward | National, urban employee scheme | Employer: 16%; Individual: 8%; total: 24% of contribution base |
| Transitional pension coefficient (τ) | Provincial implementation guidelines; Zhao et al. (2024) | 1997 reform cohort | Provincial | Typically 1.0–1.4%; set to 1.2% in baseline simulations, varying slightly by province |
| Personal account notional interest rate | PBOC benchmark deposit rates; MOHRSS annual notices | 2020–2023 | National | Based on one-year bank deposit rate plus spread; approx. 2.5–3.0% in projections |
| Fertility & migration parameters | NBS annual population sample survey | 2020–2023 | Provincial | Used for new labor force entry and interprovincial migration in population projection module; TFR assumed approx. 1.0–1.3 |
